# Mapping Psychological Well-Being in Morbid Obesity: A Network Analysis Approach

**DOI:** 10.3390/jcm14062076

**Published:** 2025-03-18

**Authors:** Giada Pietrabissa, Anna Guerrini-Usubini, Valentina Villa, Alessandro Sartorio, Gianluca Castelnuovo, Amelia Brunani

**Affiliations:** 1 Clinical Psychology Research Lab, San Giuseppe Hospital, IRCCS Istituto Auxologico Italiano, 28824 Piancavallo, Italy; g.pietrabissa@auxologico.it (G.P.); v.villa@auxologico.it (V.V.); gianluca.castelnuovo@auxologico.it (G.C.); 2 Department of Psychology, Catholic University of Milan, 20123 Milan, Italy; 3 Experimental Laboratory for Auxo-Endocrinological Research San Giuseppe Hospital, IRCCS Istituto Auxologico Italiano, 28824 Piancavallo, Italy; sartorio@auxologico.it; 4 Division of Rehabilitation Medicine, Research Laboratory in Biomechanics and Rehabilitation San Giuseppe Hospital, IRCCS Istituto Auxologico Italiano, 28824 Piancavallo, Italy; brunani@auxologico.it

**Keywords:** psychological well-being, PGWBI, morbid obesity, network analysis

## Abstract

**Background/Objectives**: Identifying key psychological well-being factors in morbid obesity is crucial for designing effective mental health and weight management interventions. This study explores the interconnections between the dimensions of psychological well-being in a large sample of adults with morbid obesity enrolled in an inpatient nutritional rehabilitation program. **Methods**: A sample of 3212 participants (F = 58%; mean age = 57.18 years; mean Body Mass Index = 43.40 kg/m^2^) completed the Psychological General Well-Being Index (PGWBI) upon admission to the clinic. A network analysis approach examined the relationships among the PGWBI dimensions (anxiety, depression, positive well-being, self-control, vitality, and general health). **Results**: Network analysis revealed that vitality and positive well-being exhibited the highest values across closeness (1.432; 0.353), strength (0.853; 0.917), and expected influence (0.853; 0917), indicating their key role in psychological well-being. Depression also demonstrated moderate relevance, suggesting its connection to other well-being factors, though it was not the primary determinant. In contrast, self-control and general health had negative strength and expected influence values (−0.660; −1.641), indicating a less central role in the network. Additionally, anxiety and depression displayed negative betweenness (−0.645), reinforcing their more peripheral position. Conversely, positive well-being and vitality showed the highest betweenness (1.291), highlighting their role as key connecting nodes within the well-being network. **Conclusions**: Findings suggest interventions targeting positive well-being and vitality may most effectively enhance psychological well-being in morbid obesity, emphasizing strength-based approaches that foster positive affect, motivation, and resilience rather than focusing solely on reducing distress or weight-related concerns.

## 1. Introduction

The prevalence of morbid obesity has steadily increased worldwide, posing significant challenges to public health and economic stability due to its substantial healthcare costs [1,2]. Defined as a Body Mass Index (BMI) ≥ 40 or ≥35 kg/m^2^ with obesity-related health conditions, this complex metabolic disorder is associated with severe comorbidities, including cardiovascular disease, type 2 diabetes, sleep apnea, osteoarthritis, and various types of cancer [3]. Furthermore, obesity is a chronic, relapsing condition that often persists despite multiple interventions [4,5,6].

Beyond its well-documented physical burdens, obesity has a profound impact on mental health. Individuals with severe obesity experience higher rates of depression [7], anxiety [8,9,10], and reduced self-esteem and overall well-being [11,12,13]. Stigmatization is also pervasive across various aspects of life [14,15,16], including interpersonal relationships [17,18,19], education, employment [20], healthcare settings [21,22], and even everyday activities, such as being able to fit into public seating [23]. This social marginalization further deteriorates psychological well-being [24]. Additionally, psychological distress can hinder adherence to dietary recommendations and behavioral changes essential for weight management [25,26]. Despite the clear connection between obesity and mental health, healthcare systems often prioritize acute medical conditions and weight loss over comprehensive well-being, leading to fragmented and insufficient care that leaves many patients dissatisfied [27,28].

Psychological well-being is a crucial construct in health research, encompassing emotional, cognitive, and social dimensions of life [29,30]. It is recognized as an independent predictor of morbidity and mortality, both in healthy individuals and clinical populations, and includes those with severe obesity [31,32,33].

Traditionally, mental health research has treated psychopathology (e.g., depression and anxiety) and subjective well-being (e.g., life satisfaction and positive emotion) as distinct domains. However, emerging evidence suggests they are interrelated and should be examined together for a more comprehensive understanding of mental health [34,35].

Existing research often explores linear relationships between psychological factors and weight-related outcomes [36]. Such approaches may overlook the intricate and dynamic interplay between these variables. Identification of the most influential aspects of psychological well-being in individuals with morbid obesity is essential for the development of targeted interventions that enhance both mental health and weight management. This calls for innovative analytical frameworks that capture these complexities [37].

Network analysis provides a novel approach by mapping interconnections between psychological dimensions, offering deeper insight into how different aspects of well-being influence each other in individuals with morbid obesity. Unlike traditional analyses, which primarily examine isolated pairwise associations, network analysis identifies central psychological components, distinguishes direct from indirect relationships, and visually represents interactions, enhancing interpretability of results [38,39]. By revealing key nodes and pathways within the psychological network, this methodology helps clarify how dimensions of well-being interact and reinforce one another.

This study applies network analysis to explore the interconnections between the dimensions of psychological well-being (anxiety, depression, positive well-being, self-control, vitality, and general health) in adults with morbid obesity enrolled in an intensive inpatient nutritional rehabilitation program. By identifying the most influential psychological factors within this population, we aim to contribute to a more nuanced understanding of well-being in the context of severe obesity and inform the design of more effective, patient-centered interventions.

## 2. Materials and Methods

### 2.1. Participants and Procedures

Participants were individuals with morbid obesity who were enrolled in a 4-week multidisciplinary nutritional rehabilitation program at the IRCCS, Istituto Auxologico Italiano in Verbania, Italy.

The program included individualized nutritional intervention, psychological support, and supervised physical activity. Each patient received a balanced, hypocaloric Mediterranean diet consisting of three meals a day with 18–20% protein, 27–30% fat (of which <8% was saturated fat), and 50–55% carbohydrates (<15% simple sugars), and 30 g of fiber from fresh vegetables. Under the supervision of a physiotherapist, they performed 60-min physiotherapy sessions twice a day comprising aerobic training, postural control exercises, and strengthening exercises. Additionally, they attended weekly individual and group psychological support sessions.

Subjects were eligible for the study if they met the following criteria: age ≥ 18 years; BMI greater than 40 kg/m^2^ or above 35 kg/m^2^ with obesity-related health conditions; ability to understand the study’s purpose and sign an informed consent for research participation; and completion of the Psychological General Well-Being Index (PGWBI) [40,41]. Exclusion criteria included the presence of mental, cognitive, or visual impairments that could interfere with completing the questionnaire.

The data used in this research were obtained from archival records. During their initial consultation with a physician, patients provided informed consent to participate in the rehabilitation program, which also included explicit approval for the use of their data for research purposes. This standard procedure ensured ethical compliance and transparency in the collection and use of patient information.

On the same day as the initial consultation, a clinical psychologist assessed the inclusion and exclusion criteria and administered the PGWBI as part of a routine clinical evaluation. Data were collected from January 2018 to December 2022. This study was approved by the Ethical Committee of Istituto Auxologico Italiano, IRCCS, in Milan, Italy (approval number: 03C020; date of approval: 18 February 2020). Research was carried out according to the Declaration of Helsinki and its advancements.

### 2.2. Measures

Weight and height were measured on the second day of admission to calculate BMI. Standing height was determined by a Harpenden Stadiometer (Holstein Limited, Crymych, Dyfed, UK). Weight was measured to the nearest 0.1 kg using an electronic scale (RoWU 150, Wunder Sa.bi., Trezzo sull’Adda, Italy). Demographics (gender and age) were also collected, and the Italian version of the PGWBI was used to measure psychological well-being.

The Psychological General Well-Being Index (PGWBI) [40,41] is a widely used self-report measure designed to assess a person’s level of subjective psychological well-being. In detail, it assesses self-representations of intrapersonal affective or emotional states reflecting a sense of subjective well-being or distress, and thus captures what we could call a subjective perception of well-being. The PGWBI consists of 22 standardized items rated on a 6-point Likert scale, with responses ranging from 0 (indicating severe distress or negative feelings) to 5 (indicating positive feelings or well-being). The tool produces a single measure of psychological well-being and also provides subscales to assess the following domains: Anxiety (5 items) assesses levels of tension, worry, and nervousness. Higher scores indicate lower anxiety and a greater sense of calm (“Have you felt tense and nervous?”). Depression (3 items) evaluates feelings of sadness, discouragement, and hopelessness. A higher score reflects a more positive mood and fewer depressive symptoms (“Have you felt downhearted and unhappy?”). Positive well-being (4 items)measures feelings of happiness, life satisfaction, and optimism. A higher score suggests a greater sense of overall well-being and emotional positivity (“Have you felt happy, satisfied with life?”). Self-control (3 items) examines an individual’s ability to regulate emotions and behavior, reflecting stability and resilience. Higher scores indicate better emotional regulation and perceived control over one’s life (“Have you felt in control of yourself?). General health (3 items) assesses self-perceived physical health and vitality, including the impact of psychological distress on overall well-being. Higher scores suggest a stronger perception of good health (“How would you rate your health status?”). Vitality (4 items) captures energy levels and fatigue, reflecting overall vigor and enthusiasm for daily activities. Higher scores indicate greater energy and lower fatigue (“Have you felt full of energy?”).

The PGWBI is structured so that higher scores reflect lower levels of psychological distress and better emotional well-being. This means that higher scores on the anxiety and depression subscales indicate lower levels of anxiety and depression, respectively (no reverse scoring required). The total score is calculated by summing the responses across all items. The possible score range is between 0 and 110, with higher scores indicating better psychological well-being. PGWBI total scores are typically categorized into four well-being levels: severe distress, 0–60; moderate distress, 61–72; no distress/marginal well-being, 73–82; and positive well-being, ≥83. These cut-off points are used to classify individuals based on their psychological well-being, with lower scores indicating higher levels of psychological distress.

The PGWBI has been widely used across the world with both clinical and nonclinical samples [42,43,44]. The Italian-adapted version, developed by Grossi and colleagues [40], has shown strong internal consistency, with Cronbach’s alpha values between 0.94 and 0.96, confirming its reliability.

Similarly, in the present study, Cronbach’s alpha coefficients demonstrated strong internal consistency across all dimensions (total score: α = 0.94, anxiety: α = 0.91, depressed mood: α = 0.90, positive well-being: α = 0.92, self-control: α = 0.89, general health: α = 0.88, and vitality: α = 0.90).

### 2.3. Statistical Analysis

Data analyses were conducted using IBM SPSS Statistics for Windows, Version 23.0 [45]. Descriptive statistics were computed to summarize participant characteristics and the dimensions of the Psychological General Well-Being Index (PGWBI), and were presented as means and standard deviations or frequencies and percentages, as appropriate.

Moreover, given the normal distribution of the variables, the relationships between the six dimensions of the PGWBI (anxiety, depression, positive well-being, self-control, general health, and vitality) were examined using Pearson’s correlation coefficients and interpreted as weak (r = 0.1–0.3), moderate (r = 0.3–0.5), or strong (r > 0.5) [46]. The strength and direction of the correlations were analyzed to identify significant associations between dimensions and corresponding *p*-values were calculated. A significance level of *p* < 0.05 was used for all statistical tests.

In addition, a psychometric network analysis was performed using JASP (Version 0.19.0) [47] to disentangle the relationships among the six dimensions of the PGWBI. The analysis was performed using a Gaussian Graphical Model (GGM) with pairwise partial correlations representing the edges (relationships) between nodes (PGWBI dimensions) [48]. To minimize the presence of spurious connections, the network model was estimated using the Graphical Least Absolute Shrinkage and Selection Operator (GLASSO) regularization algorithm [49,50,51].

This approach constrains low correlation values to zero, resulting in a sparse network, by eliminating likely spurious connections. The GLASSO algorithm employs a tuning parameter (λ) to control the sparsity of the network, where higher λ values lead to greater sparsity [49,50,52]. Then, the Extended Bayesian Information Criterion (EBIC) was employed as a model-selection criterion to identify and retrieve the most optimal network structure. A γ hyperparameter was set to 0.5 to balance sensitivity and specificity in edge detection [53].

This EBIC–GLASSO approach has been recognized for its effectiveness in accurately reconstructing true network structures [54,55] in cases where the network is inherently sparse (i.e., contains relatively few connections). This method also demonstrates high specificity, effectively preventing the estimation of non-existent edges, though its sensitivity (i.e., accuracy in detecting existing connections) can vary.

Furthermore, the stability of the network model was assessed [56] using the correlation stability coefficient (CS-coefficient). CS-coefficient values higher or equal to 0.5 indicate optimal stability and values higher than 0.25 indicate moderate stability [56,57].

In addition, centrality measures were computed, including strength, closeness, betweenness, and expected influence. Strength centrality indicates the number of edges (relationships) connected to a node. Closeness centrality measures the proximity of a node to all other nodes, reflecting its level of accessibility within the network. Betweenness centrality measures interactions between nodes, depending on the other nodes that lie on the same path [58,59,60,61,62]. Last, expected influence accounts for the sum of all positive and negative connections of a node, providing insight into its overall impact on the network.

## 3. Results

### 3.1. Characteristics of the Sample

The study included 3.212 adult inpatients diagnosed with morbid obesity, with women representing 58% of the sample (*n* = 1.863). The participants had an average age of 57.18 years (SD = 14.11) and a mean BMI of 43.40 kg/m^2^ (SD = 5.66). Descriptive statistics of the sample are shown in Table 1 below.

### 3.2. Correlation Analysis

Table 2 presents the results of a correlation analysis exploring relationships among the six variables (depression, anxiety, positive well-being, self-control, general health, and vitality) that constitute the PGWBI. All correlations are positive and significant at the 0.01 level, indicating robust relationships across dimensions.

The strongest association was observed between positive well-being and vitality (r = 0.768). In addition, positive well-being showed high correlations with depression (r = 0.757) and anxiety (r = 0.720), which were also strongly related to each other (r = 0.746).

General health showed the lowest correlation indices with all the other variables; the weakest relationship was observed with self-control (r = 0.483).

### 3.3. Psychometric Network Analysis

#### 3.3.1. Network Structure and Edge Weights

**Figure 1:**
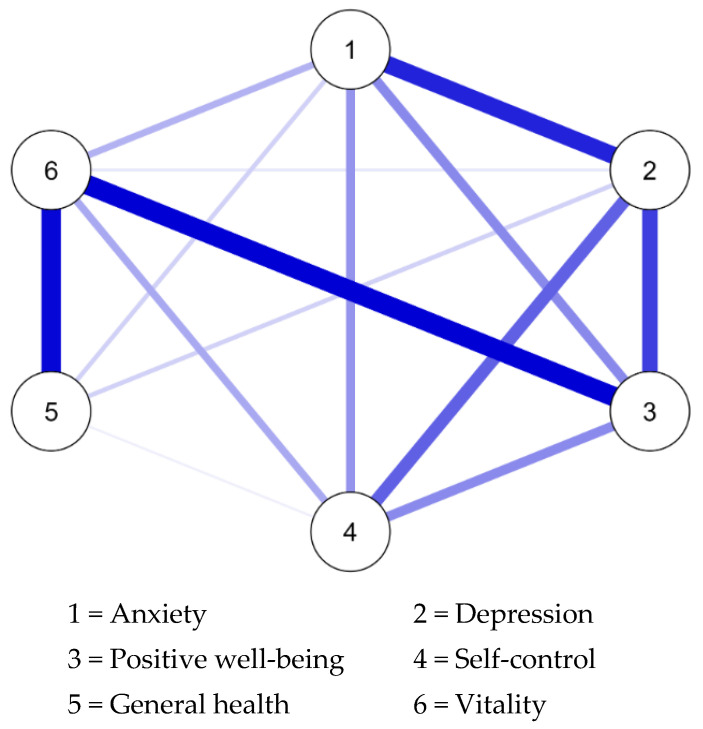
Network visualization of the PGWBI dimensions.

The network model estimated by the EBIC–GLASSO algorithm was constructed using network weights. The matrix of these weights is presented in Table 3 and includes only positive-edge weights across all connections. The strongest edge was observed between positive well-being and vitality (0.394), followed by the relationships between vitality and general health (0.385), and the edge between anxiety and depression (0.342). Moreover, a solid edge was observed between positive well-being and depression (0.298) and a moderately strong correlation was also identified between depression and self-control (0.246). Conversely, the weakest connection was found between general health and self-control (0.023), with the next weakest link being between vitality and depression (0.037). The results are reported in Table 3 and Figure 1.

#### 3.3.2. Network Stability

**Figure 2:**
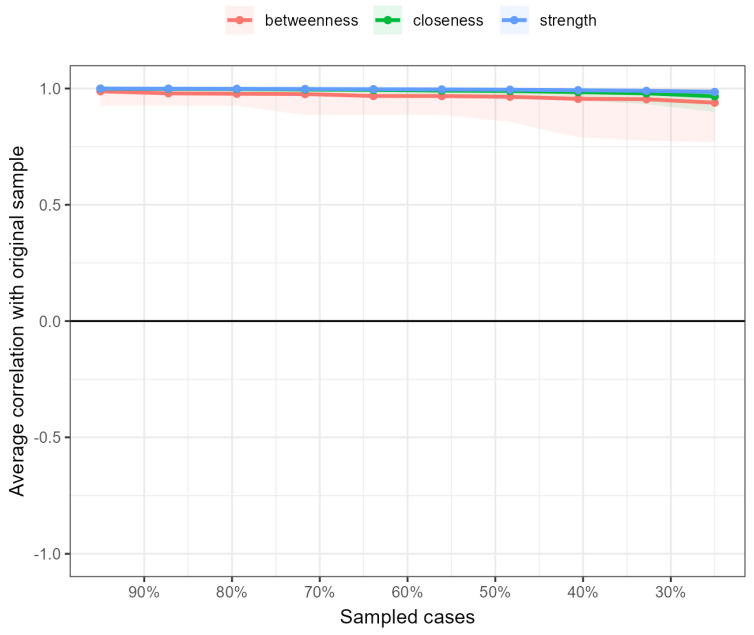
Bootstrap stability analysis of centrality measures in the network.

Stability analyses indicated that the network model had excellent stability, with a CS coefficient of 0.75 for the edges. This implies that even if up to 75% of the participants were removed from the sample, the structure of the edges would remain stable (Figure 2).

**Figure 3:**
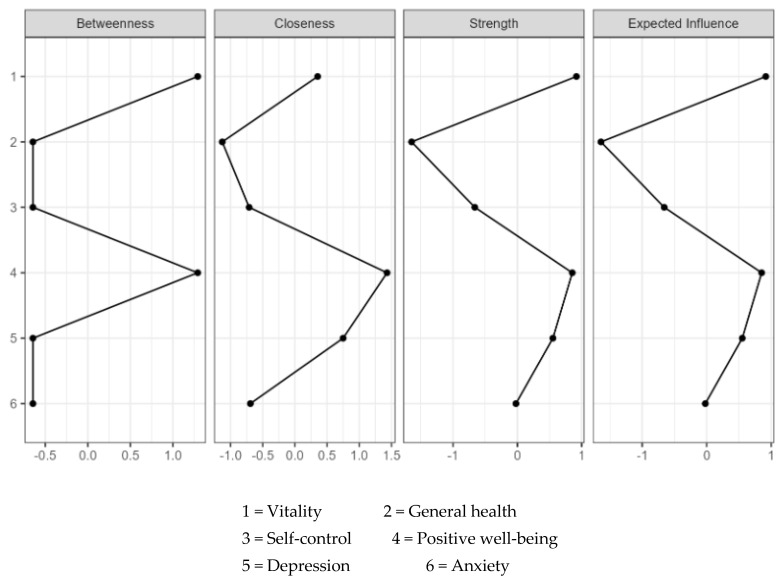
Plots of the standardized centrality indices of the network analysis.

Centrality indices indicated that vitality and positive well-being exhibited the highest values across all centrality measures, particularly in closeness, strength, and expected influence. Depression also demonstrated notable relevance, with moderate values in closeness, strength, and expected influence. In contrast, self-control and general health showed negative values for strength and expected influence. Along with anxiety and depression, they also displayed negative betweenness, suggesting a more peripheral role in the network. Moreover, positive well-being and vitality had the highest betweenness. The results are reported in Table 4 and Figure 3.

## 4. Discussion

Psychological well-being is increasingly acknowledged as a key determinant in morbid obesity, shaping both immediate and long-term health outcomes [29,63,64,65]. However, despite its well-established significance, its multifaceted nature makes it a complex and not-yet-fully-understood phenomenon. The present study aims to explore the psychological well-being of a sample of inpatients with morbid obesity through psychometric network analysis. This approach was used to reveal distinctive and potentially significant connections between different dimensions of psychological well-being assessed with the PGWBI.

A high association between the variables in this network is expected, as well-being encompasses closely related dimensions. This expectation is confirmed by the high number of non-zero edges (14 out of 15), suggesting strong interconnections among the measured constructs. Particularly noteworthy relationships emerged between positive well-being and vitality, reinforcing their theoretical and empirical link. Indeed, in the positive psychology literature, two distinct forms of positive well-being have been conceptualized: hedonic well-being and eudaimonic well-being. The first refers to positive emotions and overall life satisfaction and typically assesses the presence, frequency, and intensity of positive affect, offering insight into an individual’s subjective emotional experience. In contrast, eudaimonic well-being is associated with a sense of purpose, personal growth, and self-actualization. It reflects the emotions that arise when an individual moves toward their full potential, often assessed through indicators such as vitality, curiosity, and engagement [66]. Accordingly, the findings of the present study suggest that positive well-being and vitality exhibited the highest number of connections (strength), the greatest impact (expected influence), and were also more centrally positioned (higher closeness) within the network, thus functioning as core components of psychological well-being in individuals with morbid obesity.

These two dimensions may have differential effects on overall well-being; while positive well-being contributes to immediate emotional gratification, vitality may play a more sustained role in long-term psychological resilience and life satisfaction. This aligns with previous research indicating that positive well-being and vitality play a critical role in maintaining adherence to therapeutic programs, particularly in individuals with obesity, who often face challenges such as demotivation, stigma, and emotional distress [67,68]. Improving positive well-being could help sustain motivation and encourage active participation in treatment, thereby enhancing both psychological and physical health outcomes. Additionally, interventions that emphasize behavioral activation can boost, with wide-ranging effects, multiple aspects of psychological well-being. Indeed, vitality plays a pivotal role as a bridge connecting other dimensions within the network.

Another key result of this study is the moderate centrality of depression, which shows positive closeness, strength, and expected influence. This suggests that while depressive symptoms are connected to other well-being dimensions, they do not dominate the network. In other words, depression does not appear to be the primary driver of overall well-being in this model but, rather, interacts with other factors in a secondary role. These findings contrast with traditional models, where depression is often conceptualized as a core determinant of mental health [69,70], and suggest that reducing depressive symptoms alone may not be sufficient to enhance the overall well-being of a person. This aligns with the results of a meta-analysis of 26 studies [71] that suggest positive affect exerts a protective influence on health beyond the mere absence of emotional distress. In other words, well-being does not solely stem from the absence of negative emotions but also the presence of positive experiences and engagement [72,73,74]. These insights reinforce the importance of integrating positive psychological interventions that promote positive experiences, rather than solely reduce negative emotions, to enhance long-term well-being in individuals with morbid obesity.

Conversely, self-control and general health exhibited negative strength and expected influence values, suggesting that these dimensions play a less central role within the well-being network, potentially exerting their effects in more indirect or context-dependent ways. The peripheral position of general health is particularly noteworthy, indicating that perceived physical health may not be a primary driver of psychological well-being. These findings align with research highlighting that subjective health perception does not always directly influence psychological well-being [75,76,77] and reinforce the importance of integrating psychological interventions into obesity treatment programs, rather than focusing exclusively on weight reduction or medical management. Indeed, self-control displayed negative centrality values, suggesting that it does not function as a key integrative component within the well-being network in this population. This contrasts with studies that have highlighted the role of self-regulation in weight management and emotional well-being [78,79]. One possible explanation is that in individuals with morbid obesity, self-control may not be a primary determinant of psychological well-being, particularly if emotional distress, stigma, or learned helplessness mechanisms play a stronger role [80,81]. Moreover, the moderate connection between anxiety and depression is consistent with well-established findings that these two affective states frequently co-occur and share underlying cognitive and emotional mechanisms [82,83]. However, the network suggests that depression has stronger links to well-being factors like positive well-being and vitality, whereas anxiety appears to be less integrated into the overall well-being structure. This distinction may have implications for tailoring psychological interventions, as strategies aimed at reducing depression may also enhance well-being, whereas the impact of anxiety reduction on the broader well-being network might be less integrative than expected, possibly due to individual differences in coping mechanisms or resilience [84,85]. Interventions targeting these areas may, therefore, need a more individualized approach, rather than expecting a widespread impact on well-being.

The present study has notable strengths but also limitations that warrant discussion. The main strength of this study is the use of network analysis to investigate the interconnections between well-being dimensions in a large group of inpatients seeking treatment for morbid obesity. This methodology sheds light on how variables within the same construct interact and reinforce one another, offering deeper insight into the complex interplay between psychological processes involved in morbid obesity [38].

However, some limitations should be acknowledged. First, only one measure of psychological well-being was included. Additionally, the integration of additional variables, such as measures of eating disorders, body image, or self-efficacy, would have allowed for a more comprehensive understanding of the complex interrelationship between psychological factors in inpatients with morbid obesity. Furthermore, the inclusion of BMI would have allowed for the exploration of potential associations between psychological well-being and weight-related factors. Still, BMI is an external factor that does not function as a psychological dimension within the PGWBI structure, making it less suitable for inclusion in a network analysis designed to map relationships between psychological constructs, and should be investigated in further studies. Moreover, the cross-sectional nature of this study design hinders its ability to establish causality between relationships observed in the network and the lack of longitudinal data restricts its capacity to monitor changes in psychological well-being over time, including before and after the rehabilitation program. In addition, data were obtained from a single clinical center, requiring caution when generalizing the findings to other populations or settings. Specifically, these findings may not be generalizable to individuals with morbid obesity who are not actively seeking treatment for weight reduction and rehabilitation or to patients treated in different settings (i.e., outpatient or bariatric surgery). Future research should explore whether similar psychological well-being patterns emerge in non-treatment-seeking populations and across various clinical and community-based interventions to enhance the applicability of these results. Last, the study design does not permit us to draw conclusions about clinical implications, only to propose hypotheses that should be tested in future studies.

Despite these limitations, the findings of this study provide valuable insight for the implementation of tailored interventions targeting psychological well-being in morbid obesity.

Indeed, the strong interconnectedness of positive well-being and vitality suggests that targeting these dimensions in psychological interventions could yield widespread benefits across the well-being structure.

Depression plays a role, but it is not the primary driver of psychological well-being in this population. This underscores the importance of moving beyond a symptom-reduction model—which primarily focuses on mitigating distress—toward a broader framework that actively cultivates positive psychological states. To this aim, strategies such as positive psychology interventions [86] have been shown to reduce depressive symptoms while simultaneously enhancing overall well-being and resilience [87,88]. Additionally, exercise-based programs [89] have been associated with enhanced emotional regulation, increased self-efficacy, and reduced symptoms of depression and anxiety [90,91].

Mindfulness techniques [92], including meditation, body awareness, and acceptance-based strategies, may be particularly beneficial for individuals with obesity-related distress, as they have been found to reduce emotional distress and promote adaptive coping mechanisms [93,94]. In addition, interventions based on motivational interviewing (MI) [12,95] could further strengthen the ability to regulate behavior and emotion. MI has been widely applied in weight management and obesity treatment, demonstrating efficacy in enhancing intrinsic motivation for lifestyle changes, encouraging self-efficacy and autonomy in goal-setting, and addressing ambivalence toward behavior modification [96]. Additionally, MI techniques align with research on self-regulation and emotional competence, which suggests that greater behavioral control is associated with better emotional resilience and psychological well-being [97]. This makes MI an ideal complement to positive psychology interventions, ensuring that individuals not only experience greater positive affect and vitality, but also develop the cognitive and behavioral tools to sustain long-term improvement.

## 5. Conclusions

This is one of few studies available in the literature exploring psychological well-being in a large sample of adult inpatients with morbid obesity using a network analysis approach. Network analysis proves to be a valuable approach to personalize clinical intervention and identify critical areas for targeted action. By analyzing the interconnections among various dimensions of well-being, the findings provide valuable insight into the complex relationships that shape psychological well-being in this population. Overall, these findings highlight the dominance of positive well-being and vitality in shaping psychological health, emphasizing their importance in both theoretical models of well-being and practical intervention strategies. At the same time, the relatively peripheral roles of general health perception and anxiety suggest that well-being cannot be fully explained through physical health status or distress-related factors alone. Instead, a holistic strength-based approach that prioritizes positive emotional and behavioral engagement may be the most effective pathway to improving well-being in individuals with morbid obesity.

## Figures and Tables

**Table 1:** Descriptive statistics of the sample.

	Mean (SD)	Min–Max
Age (years)	57.18 (14.11)	19–90
BMI (kg/m^2^)	43.40 (5.66)	3500–7392
PGWBI_Depression	11.34 (3.12)	0–24
PGWBI_ Positive well-being	10.23 (4.25)	0–20
PGWBI_Self-control	10.43 (3.34)	0–19
PGWBI_ General health	8.46 (3.04)	0–21
PGWBI_Vitality	10.41 (4.24)	0–20
PGWBI_Anxiety	15.71 (5.54)	0–43
PGWBI_Total	66.57 (20.00)	2–110
Sex	Frequency	%
Male	1.349	42.0
Female	1.863	58.0

Note: PGWBI total scores cut-off point: severe distress = 0–60; moderate distress = 61–72; no distress/marginal well-being = 73–82; and positive well-being ≥83 [40,41].

**Table 2:** Correlations among the six dimensions of the PGWBI.

	DEP	ANX	PWB	SC	GH	VIT
Depression	-					
Anxiety	0.746 *	-				
Positive well-being	0.757 *	0.720 *	-			
Self-control	0.699 *	0.663 *	0.694 *	-		
General health	0.522 *	0.519 *	0.541 *	0.483 *	-	
Vitality	0.669 *	0.668 *	0.768 *	0.646 **	0.654 *	-

* The correlation is significant at the 0.01 level (two-tailed). Note: DEP = depression, ANX = anxiety, PWB = positive well-being, SC = self-control, GH = general health, and VIT = vitality.

**Table 3:** Undirected weights matrix of the EBIC–GLASSO network model.

	Anxiety	Depression	Positive Well-Being	Self-Control	General Health	Vitality
Anxiety	-					
Depression	0.342	-				
Positive well-being	0.182	0.298	-			
Self-control	0.165	0.246	0.181	-		
General health	0.071	0.071	0.000	0.023	-	
Vitality	0.119	0.037	0.394	0.133	0.385	-

**Table 4:** Centrality indices of PGWBI dimensions in the network model.

	Network			
	Betweenness	Closeness	Strength	Expected Influence
Anxiety	−0.645	−0.693	−0.022	−0.022
Depression	−0.645	0.749	0.552	0.552
Positive well-being	1.291	1.432	0.853	0.853
Self-control	−0.645	−0.712	−0.660	−0.660
General health	−0.645	−1.130	−1.641	−1.641
Vitality	1.291	0.353	0.917	0.917

## Data Availability

The data presented in this study are available on request from the corresponding author.
